# MRI for the preoperative evaluation of femoroacetabular impingement

**DOI:** 10.1007/s13244-015-0459-0

**Published:** 2015-12-29

**Authors:** Angela E. Li, Shari T. Jawetz, Harry G. Greditzer, Alissa J. Burge, Danyal H. Nawabi, Hollis G. Potter

**Affiliations:** Department of Radiology and Imaging, Hospital for Special Surgery, 535 E 70th Street, New York, NY 10021 USA; Sports Medicine and Shoulder Service, Hospital for Special Surgery, 535 E 70th Street, New York, NY 10021 USA

**Keywords:** Magnetic resonance imaging, Femoroacetabular impingement, Cartilage, Arthroscopy, Preoperative

## Abstract

Femoroacetabular impingement (FAI) refers to a condition characterized by impingement of the femoral head–neck junction against the acetabular rim, often due to underlying osseous and/or soft tissue morphological abnormalities. It is a common cause of hip pain and limited range of motion in young and middle-aged adults. Hip preservation surgery aims to correct the morphological variants seen in FAI, thereby relieving pain and improving function, and potentially preventing early osteoarthritis. The purpose of this article is to review the mechanisms of chondral and labral injury in FAI to facilitate an understanding of patterns of chondrolabral injury seen on MRI. Preoperative MRI evaluation of FAI should include assessment of osseous morphologic abnormalities, labral tears, cartilage status, and other associated compensatory injuries of the pelvis. As advanced chondral wear is the major relative contraindication for hip preservation surgery, MRI is useful in the selection of patients likely to benefit from surgery.

*Teaching points *

• *The most common anatomical osseous abnormalities predisposing to FAI include cam and pincer lesions.*

• *Morphological abnormalities, labral lesions, and cartilage status should be assessed.*

• *In cam impingement, chondral wear most commonly occurs anterosuperiorly*.

• *Pre-existing advanced osteoarthritis is the strongest predictor of poor outcomes after FAI surgery.*

• *Injury to muscles and tendons or other pelvic structures can coexist with FAI.*

## Introduction

Femoroacetabular impingement (FAI) refers to pathological contact between an abnormally shaped femoral head and acetabulum, which can result in early labral and chondral damage. It is an important cause of hip pain and restricted range of motion in young adults. Symptomatic FAI ultimately requiring surgery is more prevalent in high-level athletes than in individuals who participate in recreational sports activity [1]. FAI is the most common cause of labral tears; osseous morphological changes associated with FAI have been found in up to 79 % of patients with symptomatic tears [2]. FAI predisposes patients to premature chondral wear and osteoarthritis [3–5].

Initial treatment of FAI is typically conservative in nature, and can include activity modification, non-steroidal anti-inflammatory medications, and physical therapy. If the pain causing limitation of activity persists, surgery can be considered. Hip preservation surgery aims to treat the structural lesions causing FAI [6], thereby relieving pain and improving function. Whilst it has been proven to improve symptoms, and some studies have shown promising results in the prevention of osteoarthritis, there is insufficient data to conclude whether it prevents the onset of early osteoarthritis [7–9]. Hip preservation surgery can be arthroscopic or open, and may include acetabular rim resection, osteochondroplasty, and labral or cartilage debridement or repair techniques. Pre-existing advanced osteoarthritis is the strongest predictor of poor outcomes following FAI surgery [10–12], and thus the major relative contraindication to hip preservation surgery is severe chondral wear.

The most common anatomical osseous abnormalities predisposing to FAI include cam or pincer lesions; however, mixed cam and pincer impingement is more common than either one in isolation [7, 8]. Cam and pincer structural lesions are common, and are found in up to 25 % of asymptomatic individuals [3, 13]. As such, a diagnosis of FAI should be made only in the correct clinical context. The most common presenting symptom is activity-related groin pain which is exacerbated by hip flexion and internal rotation. Patients often have a positive impingement test, where internal rotation and adduction with the hip in 90° of flexion reproduces the pain [5, 14].

Multiple imaging modalities, including radiographs and computed tomography (CT), are used in the preoperative evaluation of FAI [15]. Radiographs often serve as an initial screening tool to assess for pincer or cam lesions, hip dysplasia, or advanced osteoarthritis [16]. In addition to findings on radiographs, CT with 3D reconstructions allows detailed assessment of osseous morphology in preoperative planning [17].

The superior soft tissue contrast of MRI [18] allows for direct evaluation of the labrum and cartilage, and can guide the selection of patients who will benefit from this surgery. MRI can be performed without or with injection of intra-articular contrast, and does not employ ionizing radiation, which is of critical importance in this young patient population. The purpose of this article is to outline the pathophysiology of FAI and to provide an approach for preoperative MRI evaluation.

## Etiology and pathophysiology

Various static and dynamic factors can contribute to abnormal biomechanics which predispose to femoroacetabular impingement [19]. Static factors include incongruence between the femoral head and acetabulum, leading to asymmetric load and mechanical stress on the chondral surfaces of the hip joint. Dynamic factors include abnormal engagement of the femoral head and acetabulum at the extremes of motion, typically in full flexion, also resulting in asymmetric load and stress on chondral surfaces [20].

Cam morphology refers to asphericity of the femoral head, with loss of offset of the femoral head–neck junction, likened to a “pistol grip” deformity as seen on anteroposterior radiograph images. The physis extends lateral to the circular region of the femoral head. One hypothesis for the development of the aspherical femoral head is abnormal growth and lateral extension of the femoral head physis, with eccentric closure [21]. It has been posited that intense physical activity in childhood can result in premature physeal arrest and the development of cam lesions [22].

**Fig. 1:**
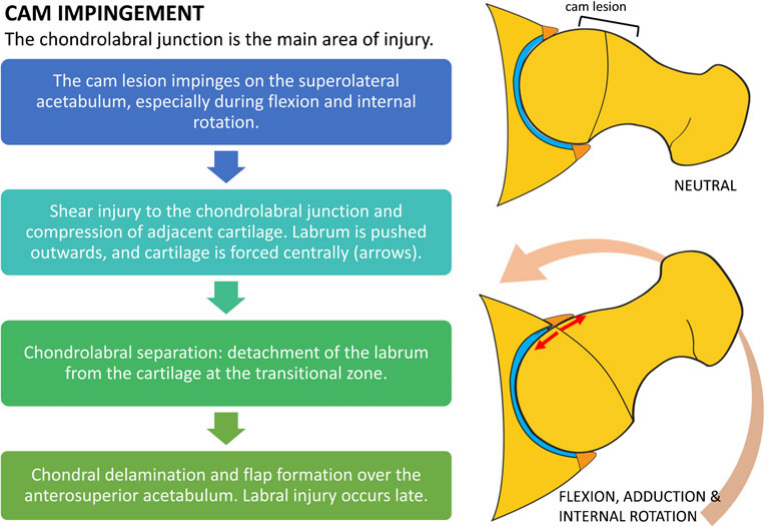
Diagram outlining the mechanisms of chondral and labral injury in cam impingement. The main focus of injury is the chondrolabral junction. There is relative sparing of the labrum until later in the disease process

Cam impingement causes shear forces at the chondrolabral junction. With hip flexion and internal rotation, the labrum is pushed outward and the cartilage is compressed and pushed centrally into the joint, initially resulting in separation of the labrum and cartilage at the transitional zone (defined as the region between the labrum and cartilage), termed chondrolabral separation or labral detachment [3, 5]. This is followed by adjacent chondral delamination near the transitional zone [3, 10] (Fig. 1). The main focus of injury, therefore, is the chondrolabral junction; intrasubstance labral tears tend to occur later in the disease process. Due to the predominantly peripheral (outer) location of labral tears in cam impingement and blood supply from the capsule, healing rates are more favourable than for labral tears in pincer impingement [4].

**Fig. 2:**
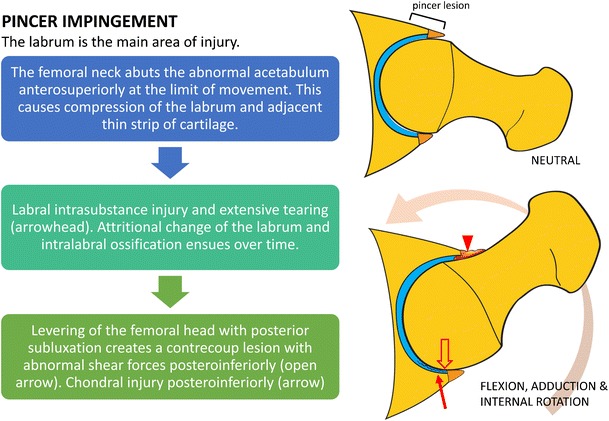
Diagram outlining the mechanisms of chondral and labral injury in pincer impingement. The labrum is the main focus of damage. Chondral injury is initially limited to a relatively small strip of cartilage at the transition zone

Pincer-type lesions are related to overcoverage of the femoral head. These are most commonly due to focal superior acetabular retroversion, but global acetabular overcoverage may also cause pincer-type impingement. Repetitive contact between the prominent acetabular rim and femoral neck during flexion and internal rotation results in labral compression. The labrum is the first structure to sustain injury, with labral degeneration and intrasubstance tears most commonly found anterosuperiorly [3, 5] (Fig. 2). Only a thin strip of cartilage adjacent to the labrum is compressed, resulting in limited chondral wear, compared to the more extensive chondral delamination or deep chondral wear seen with cam impingement [5]. Levering of the femoral head results in contrecoup injury during hip flexion and internal rotation, with chondral wear over the posteroinferior acetabulum [3].

Cam and pincer lesions are usually idiopathic in etiology, although they can be secondary to developmental abnormalities. Perthes disease and slipped capital femoral epiphysis can lead to the development of cam lesions (Table 1). Prior trauma or hip surgery can result in either cam or pincer morphology. Occurrence of pincer FAI has been reported after iatrogenic overcorrection of acetabular dysplasia with periacetabular osteotomy [23].

**Table 1:** Etiology of cam and pincer lesions

	Cam lesions	Pincer lesions
Primary	Idiopathic	Idiopathic
Secondary	• Developmental	• Developmental
	• Coxa vara	• Coxa profunda
	• Perthes disease	• Protrusio acetabuli
	• SCFE	• Traumatic
	• Traumatic	• Post-traumatic deformity of acetabulum
	• Malunited femoral neck fracture	• Iatrogenic
	• Iatrogenic	• Overcorrection of hip dysplasia
	• Femoral head osteotomy	

*SCFE* slipped capital femoral epiphysis

There are gender-specific differences in FAI, with large cam lesions more prevalent in young males and pincer lesions more common in middle-aged females [5, 24, 25]. The association between hip dysplasia and FAI is well known, with 75 % of patients with hip dysplasia having an aspherical femoral head or insufficient offset of the femoral head–neck junction, predisposing to symptomatic impingement [26].

## MR imaging technique

Variable accuracy has been reported for non-contrast MRI for the detection of labral tears, depending on the magnetic field strength, slice thickness, and field of view [27]. Three studies using high-resolution MRI at 1.5T and a small field of view demonstrated sensitivity of 77–97 % for the detection of labral tears compared with a surgical reference standard [28–30].

Direct MR arthrography involves intra-articular injection of diluted gadolinium contrast into the hip joint, and has the benefit of joint distention and improved contrast-to-noise ratio. A recent review of 19 studies evaluating MR arthrography demonstrated sensitivity of 69–100 % for the detection of labral tears with a surgical reference standard, with 12 of these studies showing sensitivity greater than 90 % [31].

A meta-analysis found that MR arthrography had higher sensitivity but lower specificity than conventional MRI for the detection of labral tears [27]. For evaluation of chondral lesions, MRI has shown higher diagnostic accuracy, with another meta-analysis demonstrating pooled sensitivity and specificity of 59 % and 94 % for MRI, compared to 62 % and 86 % for MR arthrography [32]. MR arthrography has the disadvantage of being more time consuming and invasive, with some patients experiencing post-procedural pain [33, 34]. The choice between conventional MRI and MRA will vary by institution and will depend on the ability to optimize the MRI scanning protocol with each technique.

**Fig. 3:**
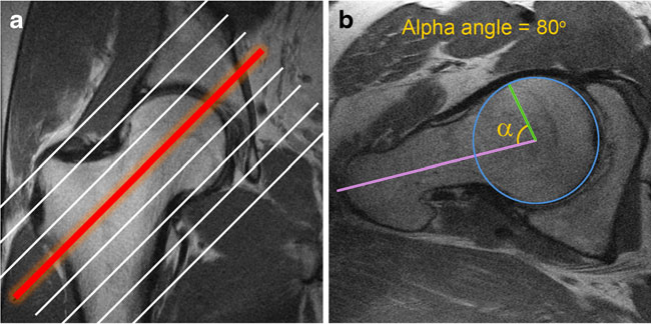
Obtaining Swiss axial images and calculating the alpha angle. **a** The Swiss axial (axial oblique) images are obtained along the long axis of the femoral neck. **b** To calculate the alpha angle, the axial oblique image through the midportion of the femoral neck (*red line*) is chosen. A circle is drawn over the femoral head cortex (*blue circle*). A line is drawn through the long axis of the femoral neck at its narrowest point (*purple line*), through the center of the femoral head. Another line is drawn from the center of the femoral neck to the point where the femoral head–neck junction meets the circle (*green line*). The alpha angle is the angle between the two lines. An alpha angle of >55° is considered a risk factor for FAI

At the authors’ institution, MRI of the hip is performed using a cardiac or small body coil on a 1.5T or 3.0T scanner. Dedicated non-contrast high-resolution fast spin-echo sequences of the affected hip are performed in the sagittal, coronal, and axial oblique planes. The axial oblique plane (Swiss axial) is obtained along the long axis of the femoral neck (Fig. 3). Larger field-of-view coronal inversion recovery and axial fast spin-echo sequences of the pelvis are performed to assess for associated injuries and other causes of hip pain, including tendinopathy and the athletic pubalgia spectrum of injuries. An axial sequence of the femoral condyles is also performed to correct for distal femoral rotation in the calculation of femoral anteversion. A suggested standard MRI pulse sequence protocol is outlined in Table 2.

**Table 2:** Suggested MRI pulse sequence protocol at 3T and (1.5T)

3T (1.5T)	Coronal STIR wide FOV	Axial PD wide FOV	Sagittal PD	Axial oblique PD	Coronal PD
TR/TE (ms)	4000/19 (4000/17)	4800/40 (4000/30)	4000/30 (3500/26)	4000/40 (4000/26)	4000/32 (4000/26)
Flip angle	180 (180)	180 (180)	180 (180)	180 (180)	180 (180)
ETL	12 (7)	12 (7)	12 (8)	12 (9)	12 (7)
RBW (kHz)	50 (32)	50 (32)	50 (32)	50 (32)	50 (32)
NEX	2 (2)	1 (2)	2 (3)	2 (3)	2 (3)
Matrix	288 × 288 (256 × 192)	512 × 256 (512 × 256)	512 × 384 (512 × 384)	512 × 320 (512 × 256)	512 × 384 (512 × 384)
FOV (cm)	38 (34)	36 (36)	19 (20)	19 (20)	19 (20)
Slice thickness (mm)/gap	5.5/0 (5.0/0)	5.0/0 (5.0/0)	2.5/0 (2.5–2.8/0)	3.0/0 (3.0/0)	3.0 (3.0/0)

*STIR* short tau inversion recovery, *PD* proton density, *TR* repetition time, *TE* echo time, *ETL* echo train length, *RBW* receiver bandwidth, *NEX* number of excitations, *FOV* field of view

Radial imaging can also be performed with image slices obtained perpendicular to the hip joint. This allows evaluation of osseous abnormalities, labral and chondral lesions, and measurement of alpha angles in different clock-face locations [35]. By convention, 12 o'clock refers to the superior hip joint, and 3 o'clock refers to the anterior aspect of the hip joint bilaterally.

## Preoperative evaluation

Preoperative evaluation of FAI on MRI should include assessment of osseous morphological abnormalities, labral lesions, cartilage status, and associated soft tissue injuries. A checklist for preoperative evaluation of FAI is given in Table 3.

**Table 3:** MRI reporting checklist for preoperative FAI evaluation

Preoperative FAI reporting checklist	
Osseous morphological abnormalities	Cam lesion Pincer lesion - Superior acetabular retroversion - True acetabular retroversion - Global acetabular overcoverage: coxa profunda or protrusio Mixed cam/pincer deformity
Labral lesions	Chondrolabral separation Labral tear or degeneration Intralabral ossification
Cartilage	Chondral delamination Chondral loss Other signs of osteoarthritis: subchondral cysts, sclerosis, osteophytes
Measurements	Alpha angle Femoral anteversion
Associated injuries	Pubic symphysis stress reaction Adductor aponeurosis injury Tendinopathy and tendon tears: hip flexors, abductors, and hamstrings.

## Osseous morphology

**Fig. 4:**
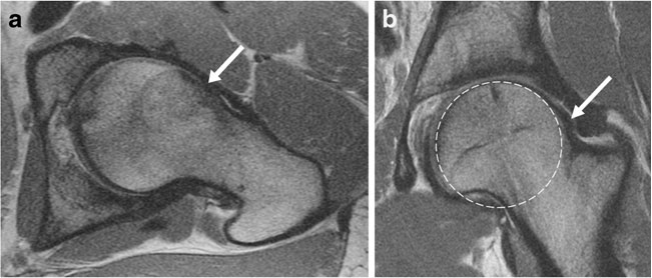
Oblique axial (**a**) and coronal (**b**) proton density (PD)-weighted MRI images in a 30-year-old man with a cam deformity. There is an osseous protuberance (*arrows*) at the femoral head–neck junction anterolaterally, with loss of offset of the femoral head–neck junction. The physeal scar extends lateral to the circular region of the femoral head (*dashed circle*) on the coronal image

The identification of osseous morphology predisposing to FAI is important, as surgical treatment of labral tears without addressing the bony impingement is a common cause for symptom recurrence [20]. Cam morphology appears on MRI as insufficient offset between the femoral head and neck, with a focal osseous bump or protuberance at the femoral head–neck junction, which can be assessed on axial or coronal images (Fig. 4). This is often associated with fibrocystic change at the femoral neck anterosuperiorly from chronic impingement, which can be seen on MRI as small cysts varying in diameter [36].

**Fig. 5:**
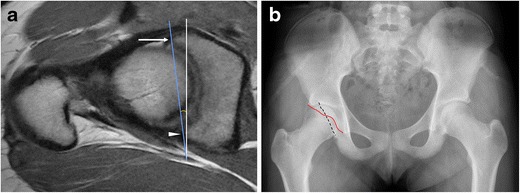
An 18-year-old girl with pincer deformity. (**a**) On an axial oblique PD-weighted MRI image superiorly, the anterior rim of the acetabulum (*arrow*) is located lateral to the posterior rim (*arrowhead*), indicative of superior acetabular retroversion (*blue line*). Superior acetabular retroversion of 8° is shown in this example. (**b**) Corresponding radiograph demonstrates the "crossover" sign where the anterior acetabular wall projects (red line) lateral to the posterior acetabular wall (black dashed line) superiorly

Pincer impingement can occur due to focal or global acetabular overcoverage. Focal acetabular overcoverage or cranial acetabular retroversion is due to an anterosuperior rim lesion, resulting in anterior acetabular overcoverage only at the superior acetabulum. The “crossover” sign may be seen on radiographs, where the anterior acetabular rim projects lateral to the posterior acetabular rim. However, a false crossover sign can appear, depending on the degree of pelvic tilt and inclination, and the tilting of the x-ray tube. Despite well-positioned radiographs, the “crossover” sign can overestimate the incidence of cranial acetabular retroversion [37]. On MRI, the degree of acetabular retroversion is determined by drawing a line between the lateral margins of the acetabulum on the cranial-most axial slices (Fig. 5).

Global acetabular overcoverage can occur due to coxa profunda, protrusio, or true acetabular retroversion, which can result in pincer impingement. True acetabular retroversion refers to posterior wall undercoverage both superiorly and inferiorly, with relative anterior acetabular overcoverage. Coxa profunda is characterized by a deep acetabulum with circumferential medial joint space loss. On radiographs, the wall of the acetabulum projects medial to the ilioischial line [38]. Acetabular depth can be calculated by measuring the distance between a line drawn through the center of the femoral head and a line joining the anterior and posterior acetabular rims [39]. Protrusio acetabuli is diagnosed when the femoral head projects medial to the ilioischial line on an AP pelvis radiograph [40].

## Labral tears and chondrolabral separation

**Fig. 6:**
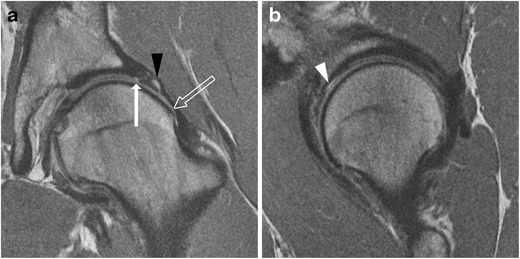
A 26-year-old man with FAI. **a** Coronal PD-weighted image shows a cam lesion at the femoral head–neck junction (*open arrow*). There is chondrolabral separation, with a cleft between the labrum and cartilage (*arrow*). A paralabral cyst is also seen (*black arrowhead*). **b** Sagittal PD-weighted image shows chondral delamination near the transition zone anterosuperiorly (*white arrowhead*)

**Fig. 7:**
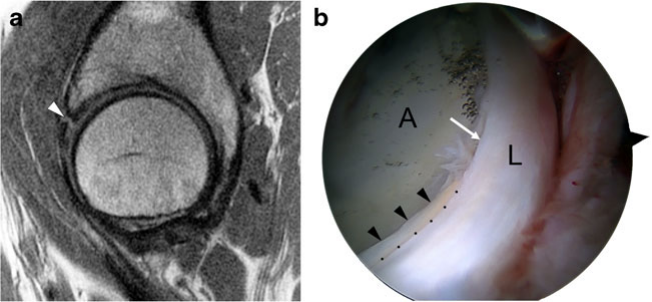
A 46-year-old woman with combined cam and pincer impingement. **a** Sagittal PD-weighted MRI demonstrates separation at the chondrolabral junction (*black arrow*). **b** Arthroscopic photo in the same patient demonstrates acetabular cartilage (A), labrum (L), and the transition zone between (*dots*). There is chondrolabral separation between 1 and 3 o'clock (*white arrow*) and chondral delamination (*black arrowheads*) adjacent to the transition zone

**Fig. 8:**
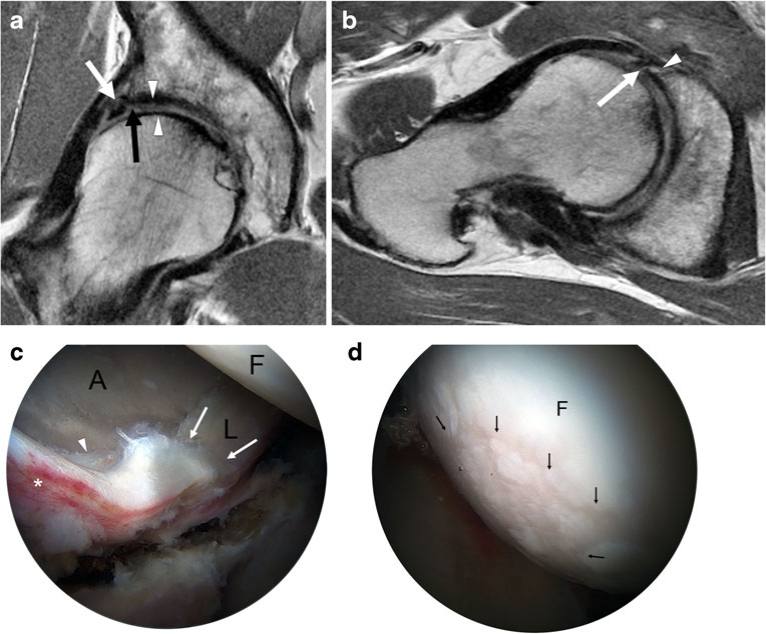
A 48-year-old man with combined cam and pincer impingement. **a** Coronal PD-weighted MRI demonstrates labral degeneration with intralabral ossification (*white arrow*). Separation is seen at the chondrolabral junction (*black arrow*). There is moderate to high-grade chondral wear over the superior femoral head and anterosuperior dome (*arrowheads*). **b** Axial oblique PD-weighted image shows a non-displaced tear of the anterior and anterosuperior labrum (*arrow*), with an associated intralabral cyst (*arrowhead*). **c** Arthroscopy image in the same patient demonstrates the labrum (L), acetabular (A), and femoral head (F) articular surfaces. An intrasubstance labral tear between 12 and 4 o'clock (*white arrows*), impaction erythema (*asterisk*), and chondral delamination at the transition zone (*arrowhead*) are seen. **d** There is moderate chondral wear (*black arrows*) over the femoral head, and normal cartilage is seen adjacent to this area (F)

The normal acetabular labrum appears as a low-signal triangle with smooth margins [41]. A common pitfall in MRI evaluation of the labrum is the presence of sublabral recesses. These are normal variants that typically do not extend the full thickness of the labrum, and are more often seen anteroinferiorly or posteroinferiorly [42, 43]. A hyperintense cleft in the anterosuperior labrum should be viewed with high suspicion for a labral tear [44]. On MRI, chondrolabral separation appears as a fluid signal intensity cleft undermining the labrum at the chondrolabral junction, with or without labral detachment (Figs. 6, 7, and 8) [15, 41].

The labral degeneration of pincer impingement appears as increased signal intensity in the labrum on fluid-sensitive MRI sequences, and it may be associated with hypertrophy of the labrum [41]. Labral tears are manifested as linear fluid signal intensity extending from the labral surface into the substance of the labrum (Fig. 8). As the disease progresses, the labrum gradually becomes thinner and increasingly attenuated until it is finally no longer visible [4]. Associated paralabral cysts are frequently demonstrated on MRI [45].

Chronic microtrauma and degeneration of the labrum results in osseous metaplasia and intralabral ossification, which is more commonly seen in pincer impingement [3]. Intralabral ossification can result in acetabular overcoverage and cause further impingement [4]. On MRI, intralabral ossification appears as small foci of signal intensity similar to bone marrow (Fig. 8) [46].

### Chondral wear and other signs of osteoarthritis

**Fig. 9:**
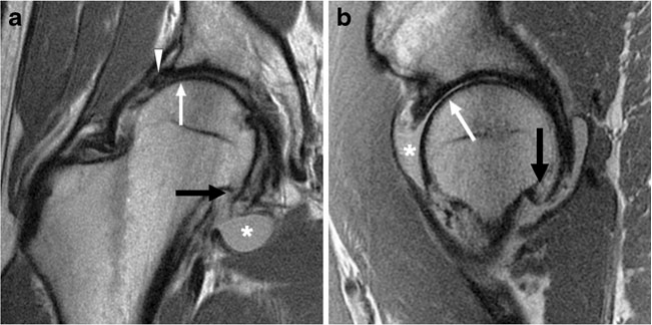
A 36-year-old man with cam-type FAI and severe osteoarthritis. Coronal (**a**) and sagittal (**b**) PD-weighted MR images of the right hip demonstrate chronic degeneration of the labrum (*arrowhead*). Chondral loss with extensive bone-on-bone contact is seen over the superior femoral head (*white arrow*). Subcapital femoral neck osteophytes are also seen (*black arrow*). Reactive synovitis with effusion is demonstrated (*asterisk*)

In cam impingement, chondral wear or delamination most commonly occurs anterosuperiorly, maximally at 1 o'clock [3, 39] (Fig. 6), appearing on the acetabular side initially, with involvement of the femoral side in more advanced cases. Pincer lesions result in greater circumferential chondral wear, which is most marked superiorly at 11 to 1 o'clock [3], or posteroinferiorly due to contrecoup forces [39]. In advanced osteoarthritis, MRI may demonstrate subchondral sclerosis, subchondral cyst formation, marginal osteophytes, or bone-on-bone contact (Fig. 9).

### Advanced cartilage imaging techniques

Assessment of cartilage on standard MRI pulse sequences is challenging due to the relatively thin layer of cartilage in the hip. Additionally, the curved surfaces of the femoral head and acetabulum result in partial volume effects, making evaluation difficult. Quantitative MRI of cartilage can detect early changes associated with chondral degeneration, and can thus provide additional information for consideration of arthroscopy or for longitudinal follow-up of patients following FAI surgery. Various quantitative MRI techniques are available, including delayed gadolinium-enhanced MRI of cartilage (dGEMRIC), and T1rho (T1_ρ_), and T2 mapping.

dGEMRIC involves intravenous administration of gadolinium contrast to assesses T1 relaxation of cartilage, requiring an injection followed by brief exercise and then scanning after 60–90 minutes. The distribution of gadolinium in the cartilage is inversely proportional to its glycosaminoglycan content. Areas of chondral degeneration with decreased glycosaminoglycan will have increased gadolinium concentration, and therefore reduced T1 relaxation times. Studies have shown that subjects with FAI have significantly lower T1 relaxation values in hip cartilage compared to asymptomatic individuals [47].

**Fig. 10:**
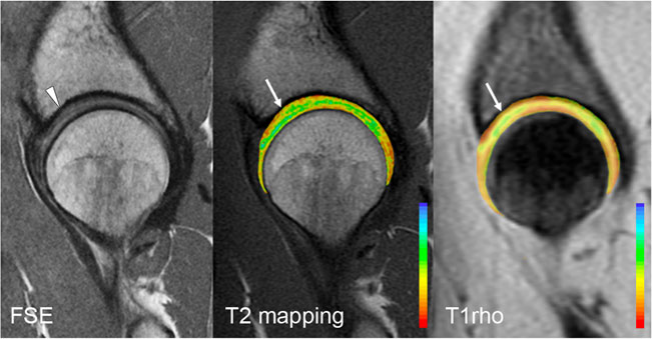
Sagittal PD-weighted MRI of the hip in a 30-year-old woman demonstrates mild chondral hyperintensity over the anterosuperior acetabular dome (*arrowhead*), with corresponding prolongation of relaxation times on T2 mapping and T1rho images (*white arrows*)

T1_ρ_ relaxation times have been correlated with proteoglycan content, and increased values of T1_ρ_ reflect proteoglycan loss [48]. T2 mapping reveals changes in collagen orientation. Disorganization of collagen occurs with cartilage degeneration, resulting in prolonged T2 relaxation times [49]. Patients with FAI have significantly higher T1rho and T2 relaxation times [50], corresponding to changes in proteoglycan and collagen content and structure (Fig. 10).

## Measurements

### Alpha angles

Alpha angles are measured utilizing oblique axial images, obtained parallel to the long axis of the femoral neck, through the midportion of the femoral neck (Fig. 3). An alpha angle of >55° is considered a risk factor for FAI [51], although there is considerable overlap between the range of alpha values in symptomatic and non-symptomatic subjects, the measured alpha angle should be considered in the clinical context of the patient’s symptoms [52].

### Femoral anteversion

**Fig. 11:**
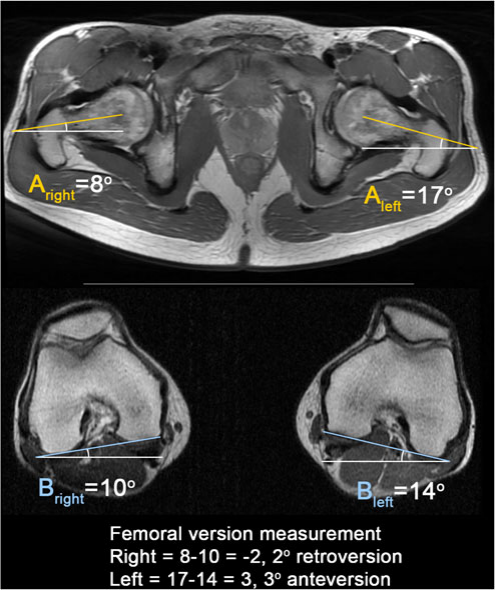
Calculation of femoral version corrected for distal femoral rotation. On the straight axial image(s) of the hip/pelvis covering the femoral head and neck, a line is drawn between the center of the femoral head and center of the femoral neck at its narrowest point to calculate the uncorrected femoral anteversion angle (A; A is a negative value when the femur is retroverted). To correct for distal femoral rotation, another line is drawn along the posterior border of the femoral condyles to calculate the angle (B; B is a negative value when the knee is internally rotated). Femoral version = A − B. A positive value indicates femoral anteversion. A negative value indicates femoral retroversion. Normal femoral anteversion is approximately 12–13°

Femoral anteversion (antetorsion) is the angle between the femoral neck and the femoral condyles. This can be calculated by measuring angles on the straight or oblique axial images of the femoral neck using a correction factor, taking into account the relative anteversion or retroversion of the femoral condyles (Fig. 11) [53]. Normal femoral anteversion is approximately 12–13° [54, 55]. Femoral retroversion or a relative decrease in femoral anteversion exacerbates the effect of a cam or pincer lesion, as impingement may occur with only minimal internal rotation and hip flexion. Increased anteversion results in reduced external rotation, with the potential for impaction of the femur on the posterior acetabulum.

## Compensatory injuries

**Fig. 12:**
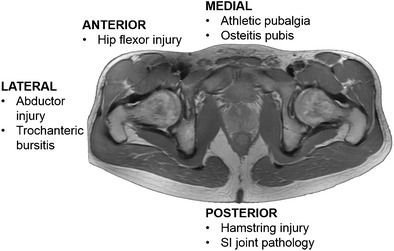
Compensatory injuries associated with FAI. Due to the altered biomechanics, there is increased strain on surrounding joints, tendons, and muscles, predisposing to injury. Preoperative MRI evaluation of FAI should include assessment of these structures

**Fig. 13:**
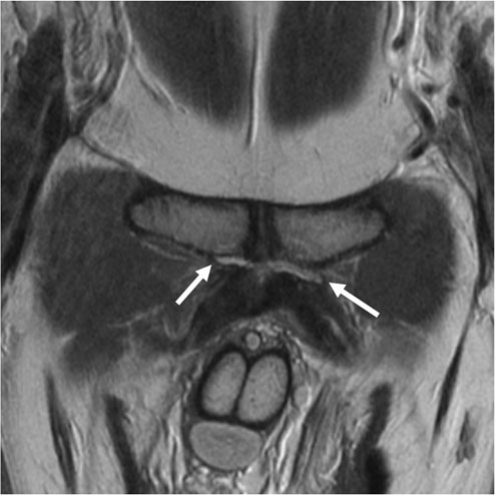
Coronal PD-weighted image of the pubic symphysis in a 34-year-old man with cam-type FAI and athletic pubalgia. There are linear fluid signal intensity clefts bilaterally, larger on the left, indicative of tears of the adductor longus tendon origins

Biomechanical alterations in the hip joint can result in abnormal forces across the pelvis and strain of other muscles and tendons [20] (Fig. 12). The athletic pubalgia spectrum of injuries often coexists with FAI. Consequently, overlapping symptomatology can make the diagnosis challenging for the clinician [56, 57]. Thus, on MRI, readers should also evaluate for features of athletic pubalgia, such as tears of the rectus abdominis-adductor aponeurosis, bone marrow edema adjacent to the pubic symphysis, or a cleft of signal hyperintensity extending from the interpubic disk along the inferomedial margin of the pubis (Fig. 13) [58]. The iliopsoas, hip abductor, and hamstring tendons should also be evaluated for concomitant tendinopathy and/or tears. The presence or absence of bursitis should be noted. Sacroiliac and lumbar spine pathology should also be excluded.

## Differential diagnoses

MRI can be used for the differential diagnosis of hip pain, including stress fractures and avascular necrosis. Other extra-articular forms of hip impingement can mimic FAI, including subspinous, ischiofemoral, and iliopsoas impingement.

Subspinous impingement occurs where a prominent anterior inferior iliac spine (AIIS) impinges against the femoral neck during flexion [59]. Enlargement or overhang of the AIIS can be developmental or due to prior avulsion or pelvic osteotomy, and can be successfully treated with arthroscopic AIIS decompression [60].

Ischiofemoral impingement involves impingement of the ischial tuberosity and the lesser trochanter, resulting in compression of the intervening quadratus femoris muscle. This is seen on MRI as narrowing of the space between the lesser trochanter and ischial tuberosity, with edema, tear, or fatty atrophy of the quadratus femoris muscle [61].

## Conclusion

Knowledge of the mechanisms of injury in FAI can facilitate an understanding of patterns of chondrolabral injury seen on MRI. As advanced chondral wear is a relative contraindication to hip preservation surgery, MRI assessment of the integrity of hip joint cartilage can assist in the selection of patients most likely to benefit from surgery. A description of the underlying osseous morphology and the presence of compensatory soft tissue injuries should also be included in the preoperative evaluation of FAI.
